# The risk of eviction and the mental health outcomes among the US adults

**DOI:** 10.1016/j.pmedr.2022.101981

**Published:** 2022-09-06

**Authors:** Binod Acharya, Dependra Bhatta, Chandra Dhakal

**Affiliations:** a Urban Health Collaborative, Dornsife School of Public Health, Drexel University, 3600 Market Street, Philadelphia, PA 19014 USA; b Behavioral and Primary Health Analytics, Northeast Delta Human Service Authority, Louisiana Department of Health, Monroe, LA 71202 USA; c Department of Agricultural and Applied Economics, University of Georgia, Athens, GA 30602 USA

**Keywords:** Mental health, Depression, Anxiety, Eviction, Housing, COVID-19

## Abstract

Although past studies establish a link between residential instability and poor mental health, studies investigating the association between perceived risk of eviction and mental health with nationally representative data are largely lacking. This study examines the association between self-reported risk of eviction and anxiety, depression, and prescription medication use for mental or emotional health reasons. This is a retrospective observational study using the repeated-cross sectional data (n = 14548; unweighted) using the US Census Bureau’s Household Pulse Survey from July 2021 to March 2022. Survey respondents aged 18 years and above who lived in rented residences and were not caught up with the rent payments at the time of the survey were included in the analysis. The descriptive summary shows a higher prevalence of depression (59.33 % vs 37.01 %), anxiety (67.01 % vs 43.28 %), and prescription medication use (26.57 % vs 23.68 %) among the respondents who are likely to face eviction in the next two months compared to the reference group not at the risk of eviction. When adjusted for demographic characteristics, family context, and socioeconomic setting, the odds of depression, anxiety, and prescription medication use in the *at-risk eviction* group were significantly higher than in the reference group. Specifically, odds ratios (ORs) [95 % CI] for depression, anxiety, and prescription medication use are 2.366 [2.364, 2.369], 2.650 [2.648, 2.653], and 1.172 [1.171, 1.174], respectively. These results suggest that the perceived risk of eviction is associated with elevated mental health problems. Addressing the housing crisis may help decrease the mental health burden among rented households.

## Highlights

• Perceived risk of eviction among people living with rent arrears is associated with elevated mental health problems.
• Prevalence of depression, anxiety, and pyschotropic medication use is higher in the at-risk of eviction group compared to the non-risk group.
• Addressing the housing crisis is crucial in decreasing the mental health burden among people living in rented residences.

## 1 Introduction

The United States is undergoing a massive housing crisis. Median housing prices have increased more than four times the rate of annual household income since 1960, accounting for inflation (Petach, 2022). The majority of low-income renting households spend over half of their income on housing (Desmond, 2018). Between 2000 and 2016; an annual average of more than 90 thousand tenants faced involuntary displacement from their residences in the form of court-ordered eviction. The US has one of the highest eviction rates among the developed countries (OECD 0000); with about one eviction filing for every 17 rented households. About half of those eviction filings result in enforced eviction upon the court order (Desmond, 2018). Eviction has been a commonplace occurrence in disadvantaged and Black neighborhoods; mostly affecting people suffering from poverty and embedded in a disadvantaged social network (Desmond and Gershenson, 2017).

A host of social and public health problems are associated with eviction. For one, poverty is often the consequence of eviction (Desmond, 2012). Evicted people struggle to get a job in the formal economy while the loss of employment and limited access to credit lending services are common, resulting in the perpetuation and reproduction of poverty (Desmond and Gershenson, 2016). They face enormous difficulty in obtaining the next housing and often have to move to neighborhoods with worse crime rates, poverty, and quality of living (Desmond and Shollenberger, 2015). Eviction is associated with poor adult self-reported health (Hatch and Yun, 2021), higher all-cause mortality (Rojas, 2017), higher substance-use-related deaths (Bradford and Bradford, 2020), increased health care expenditure (Schwartz et al., 2022), as well as frequent emergency department encounters, readmissions, and hospitalization (Navathe et al., 2018, Subedi et al., 2022). The health impacts of eviction could be inter-generational: several negative effects on childbirth and child development are associated with eviction, including a higher likelihood of pre-term birth and lower birthweight following *in-utero* exposure to eviction (Himmelstein and Desmond, 2021, Khadka et al., 2020), a higher childhood food insecurity (Leifheit et al., 2020) and poor school performance among kids (Holme,). The psychosocial and mental health consequences of housing insecurity are particularly important (Nettleton and Burrows, 2000, Libman et al., 2012, Vásquez-Vera et al., 1982, Gibson et al., 2011, Singh et al., 2019). The forced moves and housing instability are associated with developing anxiety and depressive symptoms (Alley et al., 2011, Fowler et al., 2015, Suglia et al., 2011), which can persist several years after the eviction (Desmond and Kimbro, 2015), and under extreme scenarios, could lead to suicide (Mateo-Rodríguez et al., 2019).

Although several studies suggest worsened mental health outcomes among evictees or those who are challenging the eviction filings in court (Hoke and Boen, 2021, Tsai et al., 2021), much less is known about the mental health burden experienced by people who are not evicted at the moment but consider themselves to be at a high risk of eviction in the near future. Eviction is a multi-step process starting with the landlords giving tenants notice of eviction and case filing, followed by a court hearing, order, and enforcement. The perceived threat of eviction predates the actual eviction process but individuals may experience elevated mental health challenges out of the fear of forthcoming eviction much before the eviction filings take place. Moreover, a significant proportion of involuntary displacement happens in informal ways, without the court adjudicating the case. The analyses based on the administrative eviction records, therefore, could miss an important subpopulation from the study (Desmond and Shollenberger, 2015, Lundberg and Donnelly, 2018). To address these gaps in the literature, we leverage the publicly available data from recent national surveys conducted in 2021 and 2022 and examine the association between the self-reported risk of eviction and the prevalence of depression, anxiety, and the usage of prescription medication for mental, emotional, and behavioral conditions.

Importantly, our analysis of the perceived threat of eviction and mental health corresponds to the middle of the COVID-19 pandemic, which led to massive mental distress, unemployment rate, and income decline. The people of lower socioeconomic status, who are more likely to rent the residence rather than own the house, bore the brunt of the pandemic-led economic shock (Beland et al., 2020). The economic fallout of the pandemic including, job loss, layoffs, and wage cuts exacerbated the housing situation and threatened many families out of their residences, prompting state and local governments to announce various forms of eviction moratoria. An et al. reported that implementing state-level eviction moratoria was associated with a reduction in emotional distress, with a more pronounced effect among African American people (An et al., 2021). Strong eviction moratoria that blocked the eviction process early on —such as those that prevent the landlords from giving notice of eviction and the court filling—were successful in protecting renters from mental distress (Leifheit et al., 2021). Those moratoria, albeit temporary, lowered the imminent threat of eviction and appeared to protect against mental health distress, providing indirect evidence of the relationship between the risk of eviction and mental health. We build on those studies by studying the direct measure of the perceived risk of eviction and its associations with mental health reported by renters who are not caught up with the rent payment.

## 2 Methods

### 2.1 Data source

We obtained repeated cross-sectional individual-level microdata from Household Pulse Survey (HPS), a 20-minute online, nationally representative survey conducted by the United States Census Bureau (US 0000). The survey was designed to capture the households’ social and economic experiences during the COVID-19 pandemic and has previously been used in public health research (Donnelly and Farina, 2021, Acharya and Dhakal, 2021). The survey utilized the Census Bureau’s Master Address File for selecting housing units and performed systematic sampling. We used data from the HPS survey weeks 34 to 43, spanning a timeframe from July 21, 2021, to March 14, 2022, for which the information about the perceived risk of eviction was available, and the mental-health questionnaires were asked consistently. The response rate across the survey weeks ranged from 5.4 % to 7.9 %. The HPS provides person-level survey weight to make the sample representative of the US population. Because of the design of HPS, we limit this analysis to individuals living in rented residences and not catching up with the rent payment.

### 2.2 Outcomes

We have three outcomes of interest in this study, *viz*, the exhibition of symptoms of depression, anxiety, and the usage of prescription medication for mental, emotional, or behavioral conditions. The survey asked previously validated two questions about depression (Patient Health Questionnaire (PHQ-2)) and two questions about anxiety (Generalized Anxiety Disorder questionnaire (GAD-2)) to respondents aged 18 and older. Each question had four ordinal responses, coded 0 to 1, from which respondents could choose one. A summed score of three or above on PHQ-2 and GAD-2 questionnaires would mean a clinically relevant indication of depression and anxiety, respectively (Staples et al., 2019). Following previous literature, we dichotomized the summed score such that a score of ≥ 3 in the PHQ-2 and GAD-2 questionnaire denotes a clinically relevant indication of depression and anxiety, while a score of less than 3 denote their absence (Kroenke et al., 2003, Kroenke et al., 2007). In addition, we utilized the dichotomous response question about whether a respondent took prescription medication in the last four weeks for mental, emotional, or behavioral health reasons. The survey questionnaires and possible answers are provided in Table A.1 in the supplement.

### 2.3 Exposures

The main exposure variable is the self-reported risk of eviction. When asked about the likelihood of eviction in the next two months, respondents who replied “very likely” or “somewhat likely” are considered as the *at-risk of eviction* group, while those who replied “not very likely” or “not likely at all” are considered as a non-risk group. The sampling universe for the eviction questionnaire was the people who lived in rented housing and who were not currently caught up in rent payments. Moreover, we obtained the data on the following covariates from the HPS survey: age, gender, race and ethnicity, annual household income, the highest level of education completed, number of children under 18 years of age, marriage status, indicator denoting whether the household received rental assistance, and census region of the residential addresses. The HPS categorized ethnicity as Hispanic and non-Hispanic and race as Asian alone, Black alone, White alone, and other. We collapsed Asian alone and other races in our model because of smaller samples in these two groups.

### 2.4 Statistical analysis

We performed the multivariable logistic regression analyses, separately for each of the three binary outcome variables, incorporating the survey weights. We fit three sets of models with progressive addition of covariates. In the first basic model, Model 1, the effect of eviction risk on the outcome is adjusted for the demographic characteristics of the respondents (age, sex, race/ethnicity). In Model 2, we include additional variables about family context: the marriage status of the respondents and the number of children under 18 years in the household. Finally, in Model 3, we adjust for additional variables that capture the socioeconomic context of the respondent: annual household income, the highest level of education completed, and the indicator for whether the household received rental assistance. Model 3 also adjusts for the census region and survey weeks to control for spatial and temporal fluctuations. Out of the concern that the prior mental health status might act as a confounder of the association between reported mental health status and risk of eviction, we performed sensitivity analysis by fitting additional models for depression and anxiety outcomes that adjust for all covariates in Model 3 and mental health medication use (a dummy) as a proxy of pre-existing mental health conditions. Furthermore, to address the possibility of prescription medication usage acting as a modifier of the association between perceived eviction risk and mental health, we performed sensitivity analysis by introducing an interaction between perceived eviction risk and medicine use. All models incorporate the person-level survey weight provided by HPS. Analysis was performed in SAS 9.4 and the odds ratios (ORs) are presented. Rather than reporting P-values, we deem OR to be statistically significant if the 95 % confidence interval (CI) of OR does not include 1. Drexel University IRB determined that this research is not human subjects research.

## 3 Results

The characteristics of respondents in the HPS survey by the tenure type of their residences are presented in Table A.2 in the supplement. The prevalence of depression was 18.25 % in the overall population and 30.87 % among people living in rented households. Among the renter population, a higher prevalence of depression and anxiety was present among those who were not caught up on the rent payment. Black and Hispanic people and those with less education and income were more likely to face hardship in catching up with rent payments (Table A.2 in the supplement). Our study population is the subset of the HPS respondents who live in rented residences and are not caught up with the payment. Table 1 presents the descriptive statistics of the study population by self-reported categories of eviction risk. In the study population, 42.46 % reported that they were very or somewhat likely to face eviction in the next two months. A higher proportion of individuals reported the symptoms of depression and anxiety in the *at-risk of eviction* group compared to those not threatened with eviction. A higher proportion of Black people reported being at the risk of eviction than Whites. More than 92 % of samples did not receive rental payment assistance, with a fairly similar presentation across eviction-threatened and non-threatened groups. The group at risk of eviction had lower levels of education and lower levels of household income compared to the non-risk group. About 18 % of respondents in the at-risk-of eviction group had three or more children in their households while less than 12 % of respondents in the non-risk group had 3 or more kids.

**Table 1:** Characteristics of the study population by the self-reported risk of eviction.

Characteristics	No. (%)*	
	At-risk of eviction (n = 6177)	Not at risk of eviction (n = 8371)
Prevalence of depression	3745 (59.33)	3069 (37.01)
Prevalence of anxiety	4301 (67.01)	3694 (43.28)
Users of prescription medication of mental conditions	2050 (26.57)	2425 (23.68)
Female	4343 (58.64)	5545 (57.73)
Age (years)		
18 to 24	133 (5.43)	259 (6.36)
25 to 39	1910 (38.52)	2523 (34.64)
40 to 54	2636 (36.89)	3243 (35.34)
55 to 64	1049 (13.37)	1442 (15.66)
65 and above	449 (5.79)	904 (8.00)
Race		
White	3585 (54.39)	4882 (55.28)
Black	1787 (32.87)	1860 (26.71)
Asian	179 (3.31)	884 (9.40)
Other	626 (9.43)	745 (8.61)
Ethnicity		
Hispanic	1055 (26.7)	1562 (28.02)
Non-hispanic	5122 (73.3)	6809 (71.98)
Rental assistance		
Receiver	563 (7.29)	632 (7.15)
Non-receiver	5575 (92.71)	7625 (92.85)
Education		
High school or less	1959 (61.01)	2102 (52.92)
Some college/associate degree	2964 (30.71)	3459 (30.52)
Bachelors degree	825 (5.82)	1624 (10.19)
Graduate degree	429 (2.46)	1186 (6.36)
Marital status		
Married	1543 (29.07)	2801 (36.03)
Not married	4607 (70.93)	5543 (63.97)
Number of children		
None	3025 (44.3)	4532 (48.7)
1 to 2	2271 (37.74)	2974 (39.61)
3 or more	881 (17.96)	865 (11.69)
Annual household income		
Less than $25,000	2925 (51.73)	2765 (39.69)
$25,000 - $34,999	1284 (21.39)	1670 (21.96)
$35,000 - $49,999	854 (14.39)	1323 (16.37)
$50,000 - $74,999	548 (7.57)	1099 (12.58)
$75,000 - $99,999	186 (2.98)	518 (4.98)
$100,000 and above	100 (1.95)	557 (4.43)
US Census region		
Northeast	904 (17.21)	1672 (22.99)
South	2389 (44.59)	2742 (37.05)
Midwest	1019 (15.33)	1488 (16.45)
West	1865 (22.87)	2469 (23.50)

*The numbers represent the unweighted number of respondents. The percentages are weighted by survey weights.

We present the estimated odds ratios from the models for the depression outcome in Table 2, which exhibits a positive association between eviction risk and exhibition of depressive symptoms. In Model 1, adjusted for demographic characteristics, the odds of depression were 2.485 times (95 % CI: 2.482 – 2.487) higher in the *at-risk of eviction* group compared to the group not facing the eviction threat. In Model 2, with the inclusion of family contextual variables—the marriage status and the number of children—the effect was slightly attenuated (OR: 2.470, CI: 2.467 – 2.472). In Model 3, which adjusts for additional covariates including education, income, rental assistance receipt, and fixed effects for survey weeks and census region, the odds of depression were still substantially higher among the eviction threatened group (OR:2.366, CI: 2.364 – 2.369). The parameter estimates for race/ethnicity, education attainment, and income indicated a higher depression risk among Whites, less educated compared to those with a graduate degree, and lower-income individuals compared to those with household income above $100,000. In the sensitivity analysis that controlled for all the covariates in Model 3 plus mental health medication use, the odds ratio for depression associated with eviction risk was 2.384 (CI: 2.381 – 2.386) (Table A.3 in the supplement, Column 2). Table 3 depicts the odds ratio estimates from the models for the anxiety outcome. Similar to depression, the odds of anxiety were significantly higher in the *at-risk of eviction* group compared to the non-risk group across all model specifications. In Model 1, the OR of anxiety associated with eviction risk was 2.695 and remained similar after adjusting for family contextual variables. Upon the inclusion of socioeconomic variables in Model 3, the effect of eviction risk on anxiety incidence was attenuated, but not eliminated, with the *at-risk of eviction* group much more likely to exhibit anxiety symptoms (OR: 2.650, CI: 2.648 – 2.653). The parameter estimates for race/ethnicity, education, and income were largely consistent with the estimates obtained depression model. In the sensitivity analysis that controlled for all the covariates in Model 3 plus mental health medication use, the odds ratio for anxiety associated with eviction risk was 2.685 (CI: 2.683 – 2.688) (Table A.3 in the supplement, Column 3). The prescription medication usage can act as a modifier of the association between perceived eviction risk and depression and anxiety, with that association being smaller among medicine users than non-users (Table A.4 and Table A.5 in the supplement).

**Table 2:** Adjusted odds ratios (ORs) associated with the prevalence of depression.

Variables	OR (95 % CI)		
	Model 1	Model 2	Model 3
At risk of eviction (Ref: Not at risk of eviction)	2.485 (2.482; 2.487)	2.470 (2.467; 2.472)	2.366 (2.364; 2.369)
Age, years (Ref: 18 to 24)			
25 to 39	0.865 (0.864; 0.867)	1.020 (1.018; 1.022)	1.017 (1.015; 1.019)
40 to 54	0.810 (0.809; 0.812)	0.971 (0.969; 0.973)	0.921 (0.919; 0.923)
55 to 64	0.712 (0.711; 0.714)	0.804 (0.803; 0.806)	0.774 (0.772; 0.776)
65 and above	0.515 (0.514; 0.517)	0.571 (0.569; 0.572)	0.555 (0.553; 0.556)
Female (Ref: Male)	1.028 (1.027; 1.029)	1.030 (1.029; 1.031)	1.008 (1.007; 1.009)
Race/ethnicity (Ref: NH White)			
NH Black	0.578 (0.577; 0.578)	0.566 (0.565; 0.567)	0.553 (0.552; 0.554)
Hispanic	0.577 (0.576; 0.578)	0.597 (0.596; 0.598)	0.604 (0.603; 0.604)
Other	0.533 (0.532; 0.533)	0.558 (0.557; 0.559)	0.587 (0.586; 0.588)
Married (Ref: Not married)		0.642 (0.642; 0.643)	0.678 (0.677; 0.679)
Number of children (Ref: None)			
1 to 2		0.873 (0.872; 0.874)	0.858 (0.857; 0.859)
3 or more		0.781 (0.780; 0.782)	0.724 (0.723; 0.725)
Education (Ref: Graduate degree)			
Less than high school			1.571 (1.567; 1.575)
Some college/associate degree			1.763 (1.758; 1.767)
Bachelor’s degree			1.217 (1.214; 1.221)
Income (Ref: $100,000 and above)			
Less than $25,000			1.663 (1.657; 1.668)
$25,000 - $34,999			1.362 (1.357; 1.366)
$35,000 - $49,999			1.631 (1.626; 1.637)
$50,000 - $74,999			1.726 (1.720; 1.731)
$75,000 - $99,999			1.028 (1.024; 1.032)
Region (Ref: West)			
Northeast			0.831 (0.830; 0.832)
South			0.989 (0.987; 0.990)
Midwest			0.930 (0.929; 0.932)
Survey weeks (Ref: 43)			
34			1.050 (1.048; 1.053)
35			1.154 (1.152; 1.157)
36			1.152 (1.149; 1.154)
37			1.015 (1.013; 1.017)
38			1.112 (1.109; 1.114)
39			1.127 (1.124; 1.129)
40			0.883 (0.882; 0.885)
41			0.864 (0.862; 0.866)
42			0.767 (0.765; 0.768)
Rental assistance received (Ref: not received)			0.960 (0.958; 0.961)

Notes: Model 1 includes: eviction + age + sex + race/ethnicity; Model 2 includes Model 1 + family context (marriage status + number of kids); Model 3 includes Model 2 + education + income + region + survey week + rental assistance.

**Table 3:** Adjusted odds ratios (ORs) associated with the prevalence of anxiety.

Variables	OR (95 % CI)		
	Model 1	Model 2	Model 3
At risk of eviction (Ref: Not at risk of eviction)	2.695 (2.693; 2.698)	2.695 (2.692; 2.697)	2.650 (2.648; 2.653)
Age, years (Ref: 18 to 24)			
25 to 39	0.889 (0.887; 0.891)	0.994 (0.992; 0.996)	0.987 (0.985; 0.990)
40 to 54	0.943 (0.941; 0.945)	1.066 (1.064; 1.068)	1.018 (1.016; 1.021)
55 to 64	0.745 (0.743; 0.746)	0.814 (0.812; 0.816)	0.854 (0.851; 0.856)
65 and above	0.575 (0.573; 0.576)	0.620 (0.618; 0.622)	0.595 (0.593; 0.597)
Female (Ref: Male)	1.350 (1.349; 1.351)	1.347 (1.346; 1.348)	1.316 (1.315; 1.317)
Race/ethnicity (Ref: NH White)			
NH Black	0.509 (0.509; 0.510)	0.502 (0.501; 0.503)	0.489 (0.489; 0.490)
Hispanic	0.542 (0.541; 0.542)	0.551 (0.550; 0.551)	0.546 (0.545; 0.547)
Other	0.496 (0.495; 0.497)	0.512 (0.511; 0.513)	0.488 (0.487; 0.489)
Married (Ref: Not married)		0.735 (0.734; 0.736)	0.764 (0.763; 0.764)
Number of children (Ref: None)			
1 to 2		0.953 (0.952; 0.954)	0.948 (0.947; 0.949)
3 or more		0.826 (0.825; 0.827)	0.813 (0.811; 0.814)
Education (Ref: Graduate degree)			
Less than high school			1.342 (1.338; 1.345)
Some college/associate degree			1.569 (1.565; 1.573)
Bachelors degree			1.322 (1.318; 1.326)
Income (Ref: $100,000 and above)			
Less than $25,000			1.728 (1.723; 1.733)
$25,000 - $34,999			1.487 (1.482; 1.491)
$35,000 - $49,999			1.692 (1.687; 1.698)
$50,000 - $74,999			1.689 (1.684; 1.695)
$75,000 - $99,999			1.177 (1.173; 1.182)
Region (Ref: West)			
Northeast			0.789 (0.788; 0.790)
South			0.892 (0.891; 0.893)
Midwest			0.863 (0.862; 0.865)
Survey weeks (Ref: 43)			
34			1.142 (1.139; 1.144)
35			1.021 (1.019; 1.024)
36			1.067 (1.065; 1.069)
37			1.098 (1.096; 1.101)
38			1.198 (1.196; 1.201)
39			0.943 (0.941; 0.945)
40			1.157 (1.154; 1.159)
41			0.895 (0.893; 0.897)
42			0.785 (0.783; 0.787)
Rental assistance received (Ref: not received)			0.880 (0.879; 0.882)

Notes: Model 1 includes: eviction + age + sex + race/ethnicity; Model 2 includes Model 1 + family context (marriage status + number of kids); Model 3 includes Model 2 + education + income + region + survey week + rental assistance.

In Table 4, we show the odds ratio for prescription medication use for mental health conditions. The magnitude of association between eviction risk and prescription medication use was smaller than those with depression and anxiety outcomes. Yet, we found significantly higher odds of prescription medication use among the eviction-threatened group. In the fully adjusted model, Model 3, the odds of prescription medication use were 1.171 times in the *at-risk of eviction* group compared to the non-risk group (OR: 1.172, CI: 1.171 – 1.174). Unlike in the depression and anxiety outcomes, receipt of rental assistance was positively associated with the use of prescription medication for mental health.

**Table 4:** Adjusted odds ratios (ORs) associated with the prevalence of prescription medication use for mental conditions.

Variables	OR (95 % CI)		
	Model 1	Model 2	Model 3
At risk of eviction (Ref: Not at risk of eviction)	1.187 (1.186; 1.189)	1.185 (1.184; 1.186)	1.172 (1.171; 1.174)
Age, years (Ref: 18 to 24)			
25 to 39	1.187 (1.184; 1.190)	1.296 (1.292; 1.299)	1.345 (1.341; 1.349)
40 to 54	1.479 (1.475; 1.483)	1.610 (1.606; 1.614)	1.606 (1.602; 1.611)
55 to 64	2.041 (2.035; 2.046)	2.185 (2.179; 2.192)	2.212 (2.206; 2.219)
65 and above	2.081 (2.075; 2.088)	2.193 (2.186; 2.200)	2.237 (2.230; 2.244)
Female (Ref: Male)	2.055 (2.053; 2.057)	2.059 (2.056; 2.061)	2.088 (2.086; 2.091)
Race/ethnicity (Ref: NH White)			
NH Black	0.387 (0.387; 0.388)	0.386 (0.386; 0.387)	0.396 (0.395; 0.396)
Hispanic	0.392 (0.391; 0.392)	0.400 (0.400; 0.401)	0.447 (0.446; 0.447)
Other	0.349 (0.349; 0.350)	0.357 (0.356; 0.358)	0.367 (0.366; 0.368)
Married (Ref: Not married)		0.799 (0.798; 0.800)	0.827 (0.826; 0.828)
Number of children (Ref: None)			
1 to 2		0.957 (0.956; 0.959)	0.954 (0.953; 0.955)
3 or more		0.859 (0.857; 0.860)	0.826 (0.824; 0.827)
Education (Ref: Graduate degree)			
Less than high school			0.975 (0.973; 0.978)
Some college/associate degree			1.175 (1.171; 1.178)
Bachelors degree			1.189 (1.185; 1.193)
Income (Ref: $100,000 and above)			
Less than $25,000			1.003 (1.000; 1.007)
$25,000 - $34,999			0.942 (0.939; 0.945)
$35,000 - $49,999			0.749 (0.746; 0.751)
$50,000 - $74,999			0.833 (0.830; 0.836)
$75,000 - $99,999			1.176 (1.172; 1.181)
Region (Ref: West)			
Northeast			1.017 (1.016; 1.019)
South			1.026 (1.025; 1.028)
Midwest			1.373 (1.370; 1.375)
Survey weeks (Ref: 43)			
34			1.022 (1.019; 1.025)
35			1.120 (1.117; 1.123)
36			1.169 (1.166; 1.172)
37			1.021 (1.019; 1.024)
38			1.216 (1.213; 1.220)
39			0.883 (0.881; 0.886)
40			1.168 (1.166; 1.171)
41			1.064 (1.061; 1.067)
42			1.076 (1.073; 1.078)
Rental assistance received (Ref: not received)			1.181 (1.179; 1.184)

Notes: Model 1 includes: eviction + age + sex + race/ethnicity; Model 2 includes Model 1 + family context (marriage status + number of kids); Model 3 includes Model 2 + education + income + region + survey week + rental assistance.

## 4 Discussion

This study documents the strong association between perceived eviction risk and mental health outcomes among US adults living in rented households and facing hardship in paying rent. The odds of depression, anxiety, and prescription medication use for mental health conditions in the *at-risk of eviction* group were 2.37, 2.65, and 1.17 times than those in the non-risk group, even after adjusting the models for demographic, family contextual, and socioeconomic variables. The addition of family context and socioeconomic variables attenuated but did not eliminate the effect of eviction risk on all three outcomes examined. Results from sensitivity analysis obtained after adjustment for medication use as the marker of pre-existing mental health conditions reinforce the similar magnitude of association between the risk of eviction and depression and anxiety.

During the period of the COVID-19 pandemic, when there is already a notable decline in mental health across the general population (Czeisler et al., 2020, Czeisler et al., 2021), our study indicates that vulnerable tenants living with the threat of eviction are facing an additional burden of mental health deterioration. With the fresh concerns of rising rent, inflation, and economic shocks (Lansing et al., 2022), the risk of eviction is likely to rise, compounding the mental health challenges in the country, particularly among the poor living on rent. We also found that mental health outcomes are worse among persons with lower levels of income and education compared to those with an annual household income above $100,000 and possessing a graduate degree. This is largely consistent with the past literature that documents that people in lower socioeconomic strata suffer from poor physical and mental health than their more affluent peers (Kawachi et al., 2010, Warren, 2009). Race/ethnicity was also a significant predictor of mental health outcomes, with White people having worse mental health outcomes than Black people. The lower prevalence of depression among Black people likely results from greater social support and a higher level of resilience among African American communities (Williams et al., 2007, Shim et al., 2012).

Because of the cross-sectional nature of the data and methodology used, our study is not equipped to establish the risk of eviction as a cause of mental health deterioration, but we note that the risk of eviction may affect mental health through several, possibly interconnected, channels. People living with rent arrears might consider their inability to catch up with the payment as a personal failure and a concealable stigma, eventually leading to mental health problems (Vásquez-Vera et al., 1982, Keene et al., 2015). Evictees face numerous challenges including the risk of being homeless. Evicted households often move to sub-standard housing in impoverished neighborhoods with higher health hazards, environmental exposures, and a high risk of violence (Desmond, 2012, Desmond and Shollenberger, 2015, Desmond and Kimbro, 2015). Eviction often creates an additional financial burden, for instance in the form of relocation costs and security deposits for new housing, and relocation to a new neighborhood might be a stressful event involving administrative hassles, disruption of existing social services, and constant adjustments to a new environment. People at the risk of eviction likely fear these possible trajectories of their lives, leading to an erosion of mental and emotional health.

There is an increasing recognition that eviction should be viewed through the lens of multi-dimensional poverty where a multitude of contextual and institutional factors interact among themselves to shape the eviction likelihood and that improving residential security is central to promoting public health (Alkire and Foster, 2011). If the associations we present in this study are causal, it speaks to the importance of promoting housing stability policies and programs to protect against mental health distress. Past studies indicate that housing affordability programs have lowered the eviction rate and also improved health. Policies like Medicaid expansion appear to have appreciably reduced the eviction threat by lowering the financial burden among low-income households (Zewde et al., 2019, Allen et al., 2019). A recent study indicates that states with strong economic security policies generally had a lower prevalence of depression than states without protective policies in effect (Donnelly and Farina, 2021). While specific programs like the provision of legal counsel in eviction court are associated with the decline in the eviction rate (Greiner et al., 2012, Seron et al., 2001), ending eviction probably requires policy efforts aimed at addressing structural problems with housing unaffordability. Targeted policies such as the enforcement of eviction moratoria during the COVID-19 can shield struggling renters from eviction and protect against mental health decline (Leifheit et al., 2021). In addition, healthcare institutions and hospital systems can potentially invest in housing resources and other forms of assistance that might be valuable in improving mental health.

While eviction as a cause of mental health decline is widely discussed in the literature, there remains the possibility of reciprocal association—that poor mental health could be a reason behind the increased risk of eviction (Libman et al., 2012, Schwartz et al., 2021). This reciprocal relationship between rent delinquency and mental health is possible because people with mental illnesses might struggle to make a stable income, putting them behind in rent payments and increasing the likelihood of eviction. While future studies specifically designed to answer these causal questions can inform about the direction of the relationship, the results from this study will be still valuable in understanding the interplay between the risk of eviction and mental health. For instance, if the reciprocal associations hold, it would suggest that people with poor mental health are at an increased risk of eviction. This highlights the importance of prioritizing mental health care that not only promotes community health but also promotes residential stability. In other words, public policies that incorporate the co-constitutive relationship between mental health and eviction are important in improving population health as well as housing security.

## 5 Limitation

We lacked the residential addresses of the survey samples, which limited our ability to examine the impact of neighborhood-level factors such as chronic stressors in the neighborhoods that may affect or mediate mental health outcomes (Silver et al., 1982, Matheson et al., 2006, Hill et al., 2005). Furthermore, the HPS questionnaire about the risk of eviction was not asked for all persons living in rented residences but was asked only for those tenants who were not catching up with the rent payments at the time of the survey. Because the sampling universe in this study is already limited to those who were having difficulty in making timely rent payments, our results could be an overestimation compared to the results that would have been obtained by including all rented households. We also acknowledge the relatively low response rate of the HPS survey which ranged from 5.4 % to 7.9 %. This study focuses on the general renting population that is already behind on rent and we do not perform sub-group/interaction analyses, although the differential prevalence of mental health problems and eviction risk across various groups are possible. Moreover, the association between the risk of eviction and mental health reported in this paper does not necessarily imply a causal relationship because of the possibility of reverse causality. Finally, we emphasize that the depression and anxiety determination in the study are not clinical diagnoses made by the health care provider but are based on responses to PHQ-2 and GAD-2 questionnaires, which might warrant caution in interpretation.

## 6 Conclusions

We found evidence that the risk of eviction is associated with the clinically meaningful exhibition of depression and anxiety symptoms, and prescription medication use for mental or behavioral health conditions. Housing policies and programs protecting tenants from eviction risk are important in improving the mental health of US adults.

Funding

This research did not receive any specific grant from funding agencies in the public, commercial, or not-for-profit sectors.

## CRediT authorship contribution statement

**Binod Acharya:** Conceptualization, Methodology, Software, Writing – original draft, Writing – review & editing. **Dependra Bhatta:** Methodology, Investigation, Writing – review & editing. **Chandra Dhakal:** Methodology, Investigation, Writing – review & editing.

## Declaration of Competing Interest

The authors declare that they have no known competing financial interests or personal relationships that could have appeared to influence the work reported in this paper.

## Figures and Tables

**Table A.1:** Survey questions from the household pulse survey (HPS) related to outcome and primary exposure variables.

Category	Question	Possible answers*
Outcome variables		
Depression (PHQ-2)	Over the last 2 weeks, how often have you been bothered by feeling down, depressed, or hopeless? Select only one answer.	a) Not at all (0)b) Several days (1)c) More than half the days (2)d) Nearly every day (3)
	Over the last 2 weeks, how often have you been bothered by having little interest or pleasure in doing things? Select only one answer.	a) Not at all (0)b) Several days(1)c) More than half the days (2)d) Nearly every day (3)
Anxiety (GAD-2)	Over the last 2 weeks, how often have you been bothered by feeling nervous, anxious, or on edge? Select only one answer.	a) Not at all (0)b) Several days (1)c) More than half the days (2)d) Nearly every day(3)
	Over the last 2 weeks, how often have you been bothered by not being able to stop or control worrying? Select only one answer	a) Not at all (0)b) Several days (1)c) More than half the days (2)d) Nearly every day (3)
Prescription medication use	At any time in the last 4 weeks, did you take prescription medication to help you with any emotions or with your concentration, behavior or mental health? Select only one answer.	a) Yesb) No
Primary exposure variable		
Eviction	How likely is it that your household will have to leave this home or apartment within the next two months because of eviction? Select only one answer.	a) Very likelyb) Somewhat likelyc) Not very likelyd) Not likely at all

*Numbers in the parenthesis are authors’ coding.

**Table A.2:** Characteristics of respondents from household pulse survey (HPS). The values represent percentages, weighted by survey weights.

Characteristics	All*(n=659,071)	Living in rented residence (n=131,734)	Living in rent and caught-up on rent payment(n=116,329)	Living in rent and not caught up on rent payment (n=15,014)
Prevalence of depression	18.25	30.87	28.07	46.38
Prevalence of anxiety	22.74	36.94	33.99	53.05
Users of prescription medication of mental conditions	21.95	23.43	23.43	24.54
Female	51.66	54.36	53.73	57.81
Age (years)				
18 to 24	7.13	8.56	8.95	6.40
25 to 39	26.03	39.73	40.50	35.85
40 to 54	25.96	25.91	24.18	35.46
55 to 64	17.33	13.15	12.90	14.62
65 and above	23.55	12.66	13.47	7.67
Race				
White	76.51	69.29	72.07	54.55
Black	12.21	18.05	15.89	29.56
Asian	6.00	5.69	5.41	7.04
Other	5.28	6.97	6.64	8.85
Ethnicity				
Hispanic	16.97	22.96	22.16	27.38
Non-hispanic	83.03	77.04	77.84	72.62
Education				
High school or less	38.52	43.48	40.92	57.07
Some college/associate degree	30.35	31.09	31.31	30.14
Bachelors degree	17.28	15.68	17.08	8.16
Graduate degree	13.86	9.75	10.69	4.62
Marital status				
Married	55.62	35.25	35.70	32.90
Not married	44.38	64.75	35.70	67.10
Number of children in household				
None	61.69	62.02	64.77	47.04
1 to 2	30.17	29.22	27.49	38.68
3 or more	8.13	8.76	7.74	14.28
Annual household income				
Less than $25,000	15.06	28.88	25.95	45.32
$25,000 - $34,999	11.34	17.62	16.90	21.68
$35,000 - $49,999	12.39	15.32	15.32	15.39
$50,000 - $74,999	17.45	16.22	17.28	10.26
$75,000 - $99,999	13.16	9.01	9.89	4.05
$100,000 and above	30.60	12.95	14.67	3.30
US Census region				
Northeast	17.11	17.77	17.17	20.95
South	38.41	36.12	35.41	39.83
Midwest	20.56	17.04	17.29	15.81
West	23.92	29.07	30.12	23.42

*Includes all respondents irrespective the tenure type of their residence and includes owned (with or without mortgage), rented, or occupied residences without paying rent.

**Table A.3:** Adjusted odds ratios (ORs) associated with the prevalence of depression and anxiety from the sensitivity analysis.

Variables	OR (95% CI)	
	Depression outcome	Anxiety outcome
At risk of eviction (Ref: Not at risk of eviction)	2.384 (2.381 ; 2.386)	2.685 (2.683 ; 2.688)
Medication use (Ref: No)	2.712 (2.709 ; 2.716)	2.848 (2.845 ; 2.852)
Age, years (Ref: 18 to 24)		
25 to 39	0.957 (0.955 ; 0.959)	0.929 (0.927 ; 0.931)
40 to 54	0.835 (0.833 ; 0.837)	0.924 (0.922 ; 0.926)
55 to 64	0.660 (0.659 ; 0.662)	0.728 (0.727 ; 0.730)
65 and above	0.460 (0.458 ; 0.461)	0.495 (0.494 ; 0.496)
Female (Ref: Male)	0.891 (0.890 ; 0.892)	1.171 (1.170 ; 1.172)
Race/ethnicity (Ref: NH White)		
NH Black	0.649 (0.648 ; 0.650)	0.571 (0.570 ; 0.571)
Hispanic	0.694 (0.693 ; 0.695)	0.626 (0.625 ; 0.627)
Other	0.692 (0.691 ; 0.693)	0.573 (0.572 ; 0.574)
Married (Ref: Not married)	0.694 (0.693 ; 0.695)	0.786 (0.785 ; 0.786)
Number of children (Ref: None)		
1-2	0.862 (0.861 ; 0.863)	0.958 (0.957 ; 0.959)
3 or more	0.741 (0.739 ; 0.742)	0.836 (0.835 ; 0.837)
Education (Ref: Graduate degree)		
Less than high school	1.592 (1.588 ; 1.596)	1.344 (1.340 ; 1.347)
Some college/associate degree	1.749 (1.744 ; 1.754)	1.550 (1.545 ; 1.554)
Bachelor’s degree	1.187 (1.183 ; 1.191)	1.292 (1.288 ; 1.296)
Income (Ref: $100,000 and above)		
Less than $25,000	1.707 (1.701 ; 1.712)	1.809 (1.804 ; 1.815)
$25,000 - $34,999	1.404 (1.400 ; 1.409)	1.577 (1.572 ; 1.582)
$35,000 - $49,999	1.753 (1.747 ; 1.759)	1.860 (1.854 ; 1.866)
$50,000 - $74,999	1.832 (1.826 ; 1.838)	1.829 (1.823 ; 1.835)
$75,000 - $99,999	0.994 (0.990 ; 0.998)	1.167 (1.163 ; 1.171)
Region (Ref: West)		
Northeast	0.820 (0.819 ; 0.821)	0.783 (0.782 ; 0.784)
South	0.991 (0.990 ; 0.992)	0.891 (0.890 ; 0.892)
Midwest	0.874 (0.873 ; 0.876)	0.802 (0.801 ; 0.804)
Survey weeks (Ref: 43)		
34	1.049 (1.047 ; 1.052)	1.145 (1.142 ; 1.148)
35	1.138 (1.135 ; 1.140)	1.000 (0.998 ; 1.003)
36	1.137 (1.135 ; 1.140)	1.052 (1.049 ; 1.054)
37	1.011 (1.009 ; 1.014)	1.108 (1.105 ; 1.110)
38	1.083 (1.080 ; 1.085)	1.156 (1.154 ; 1.159)
39	1.156 (1.153 ; 1.158)	0.960 (0.958 ; 0.963)
40	0.852 (0.850 ; 0.854)	1.132 (1.130 ; 1.135)
41	0.854 (0.853 ; 0.856)	0.887 (0.885 ; 0.889)
42	0.756 (0.755 ; 0.758)	0.776 (0.774 ; 0.777)
Rental assistance received (Ref: not received)	0.925 (0.923 ; 0.926)	0.844 (0.842 ; 0.846)

Notes: Covariates in the model includes: eviction+age+sex+race/ethnicity+ marriage status+ number of kids)+education+income+region+survey week+rental assistance+medication use

**Table A.4:** Regression coefficients (β) from the interaction model for depression and anxiety outcomes from the sensitivity analysis.

Variables	Depression		Anxiety	
	β (95 % CI)	*P*	β (95 % CI)	*P*
At risk of eviction (Ref: Not at risk of eviction)	0.909 (0.908 ; 0.910)	<.001	1.066 (1.065 ; 1.067)	<.001
Medication use (Ref: No)	1.069 (1.067 ; 1.071)	<.001	1.192 (1.191 ; 1.194)	<.001
Eviction risk × Medication use	-0.166 (-0.169 ; -0.164)	<.001	-0.371 (-0.374 ; -0.369)	<.001
Age, years (Ref: 18 to 24)				
25 to 39	-0.042 (-0.044 ; -0.039)	<.001	-0.069 (-0.071 ; -0.066)	<.001
40 to 54	-0.178 (-0.180 ; -0.176)	<.001	-0.074 (-0.076 ; -0.071)	<.001
55 to 64	-0.412 (-0.415 ; -0.410)	<.001	-0.312 (-0.314 ; -0.309)	<.001
65 and above	-0.773 (-0.776 ; -0.770)	<.001	-0.696 (-0.699 ; -0.693)	<.001
Female (Ref: Male)	-0.116 (-0.117 ; -0.115)	<.001	0.157 (0.156 ; 0.158)	<.001
Race/ethnicity (Ref: NH White)				
NH Black	-0.433 (-0.434 ; -0.432)	<.001	-0.562 (-0.563 ; -0.561)	<.001
Hispanic	-0.365 (-0.367 ; -0.364)	<.001	-0.469 (-0.471 ; -0.468)	<.001
Other	-0.365 (-0.367 ; -0.363)	<.001	-0.553 (-0.554 ; -0.551)	<.001
Married (Ref: Not married)	-0.363 (-0.365 ; -0.362)	<.001	-0.238 (-0.239 ; -0.237)	<.001
Number of children (Ref: None)				
1-2	-0.148 (-0.149 ; -0.147)	<.001	-0.043 (-0.044 ; -0.042)	<.001
3 or more	-0.302 (-0.303 ; -0.300)	<.001	-0.183 (-0.184 ; -0.181)	<.001
Education (Ref: Graduate degree)				
Less than high school	0.464 (0.461 ; 0.467)	<.001	0.294 (0.291 ; 0.296)	<.001
Some college/associate degree	0.560 (0.557 ; 0.562)	<.001	0.440 (0.437 ; 0.442)	<.001
Bachelor’s degree	0.174 (0.171 ; 0.177)	<.001	0.262 (0.259 ; 0.265)	<.001
Income (Ref: $100,000 and above)				
Less than $25,000	0.535 (0.531 ; 0.538)	<.001	0.592 (0.589 ; 0.596)	<.001
$25,000 - $34,999	0.339 (0.335 ; 0.342)	<.001	0.453 (0.450 ; 0.456)	<.001
$35,000 - $49,999	0.562 (0.558 ; 0.565)	<.001	0.621 (0.617 ; 0.624)	<.001
$50,000 - $74,999	0.607 (0.603 ; 0.610)	<.001	0.606 (0.603 ; 0.609)	<.001
$75,000 - $99,999	-0.008 (-0.012 ; -0.004)	<.001	0.150 (0.146 ; 0.153)	<.001
Region (Ref: West)				
Northeast	-0.197 (-0.198 ; -0.195)	<.001	-0.241 (-0.243 ; -0.240)	<.001
South	-0.008 (-0.010 ; -0.007)	<.001	-0.114 (-0.116 ; -0.113)	<.001
Midwest	-0.136 (-0.138 ; -0.135)	<.001	-0.225 (-0.227 ; -0.223)	<.001
Survey weeks (Ref: 43)				
34	0.046 (0.043 ; 0.048)	<.001	0.131 (0.129 ; 0.133)	<.001
35	0.127 (0.124 ; 0.129)	<.001	-0.004 (-0.007 ; -0.002)	<.001
36	0.130 (0.128 ; 0.133)	<.001	0.054 (0.052 ; 0.056)	<.001
37	0.015 (0.013 ; 0.017)	<.001	0.110 (0.108 ; 0.112)	<.001
38	0.079 (0.077 ; 0.081)	<.001	0.145 (0.142 ; 0.147)	<.001
39	0.146 (0.144 ; 0.148)	<.001	-0.038 (-0.041 ; -0.036)	<.001
40	-0.159 (-0.161 ; -0.157)	<.001	0.126 (0.124 ; 0.128)	<.001
41	-0.158 (-0.161 ; -0.156)	<.001	-0.122 (-0.124 ; -0.120)	<.001
42	-0.279 (-0.281 ; -0.277)	<.001	-0.253 (-0.256 ; -0.251)	<.001
Rental assistance received (Ref: not received)	-0.077 (-0.079 ; -0.076)	<.001	-0.167 (-0.169 ; -0.166)	<.001
Intercept	-0.859 (-0.864 ; -0.855)	<.001	-0.720 (-0.724 ; -0.715)	<.001

Notes: Covariates in the multivariable logistic model includes: eviction+ medication use+eviction×medication use+ age+sex+race/ethnicity+ marriage status+ number of kids)+education+income+region+survey week+rental assistance.

**Table A.5:** Adjusted odds ratios (95% CI) associated with risk of eviction among prescription medication users and non-users on depression and anxiety outcome from interaction model from the sensitivity analysis.

	Depression	Anxiety
In Medicine users	2.102 (2.097 ; 2.106)	2.003 (1.999 ; 2.008)
In Medicine non-users	2.482 (2.479 ; 2.485)	2.904 (2.900 ; 2.907)

Notes: Covariates in the multivariable logistic model were: eviction+ medication use+eviction×medication use+ age+sex+race/ethnicity+ marriage status+ number of kids)+education+income+region+survey week+rental assistance.

## Data Availability

Data will be made available on request.
